# Accelerating *in Situ* Endothelialisation of Cardiovascular Bypass Grafts

**DOI:** 10.3390/ijms16010597

**Published:** 2014-12-29

**Authors:** Ee Teng Goh, Eleanor Wong, Yasmin Farhatnia, Aaron Tan, Alexander M. Seifalian

**Affiliations:** 1 Centre for Nanotechnology & Regenerative Medicine, Research Department of Nanotechnology, UCL Division of Surgery & Interventional Science, University College London (UCL), London NW3 2QG, UK; E-Mails: ee.goh.12@ucl.ac.uk (E.T.G.); eleanor.wong.12@ucl.ac.uk (E.W.); y.rafiei@ucl.ac.uk (Y.F.); aaron.tan@ucl.ac.uk (A.T.); 2 UCL Medical School, University College London (UCL), London WC1E 6DE, UK; 3 Biomaterials & Advanced Drug Delivery Laboratory, Stanford University School of Medicine, Stanford University, Palo Alto, CA 94304, USA; 4 University Department of Surgery, Royal Free London NHS Foundation Trust Hospital, London NW3 2QG, UK

**Keywords:** endothelialization, endothelial progenitor cells, bioengineered stents, nanotechnology, cardiology

## Abstract

The patency of synthetic cardiovascular grafts in the long run is synonymous with their ability to inhibit the processes of intimal hyperplasia, thrombosis and calcification. In the human body, the endothelium of blood vessels exhibits characteristics that inhibit such processes. As such it is not surprising that research in tissue engineering is directed towards replicating the functionality of the natural endothelium in cardiovascular grafts. This can be done either by seeding the endothelium within the lumen of the grafts prior to implantation or by designing the graft such that *in situ* endothelialisation takes place after implantation. Due to certain difficulties identified with *in vitro* endothelialisation, *in situ* endothelialisation, which will be the focus of this article, has garnered interest in the last years. To promote *in situ* endothelialisation, the following aspects can be taken into account: (1) Endothelial progenital cell mobilization, adhesion and proliferation; (2) Regulating differentiation of progenitor cells to mature endothelium; (3) Preventing thrombogenesis and inflammation during endothelialisation. This article aims to review and compile recent developments to promote the *in situ* endothelialisation of cardiovascular grafts and subsequently improve their patency, which can also have widespread implications in the field of tissue engineering.

## 1. Introduction

The leading cause of death and morbidity in the world is coronary artery disease [1]. In minor cases, the most common options are angioplasty and stenting. Cases where the patients have severe narrowing or blockage of the left main coronary artery or those with multiple blockages require bypass surgery [2]. While autologous transplantation of vessels such as the saphenous vein or radial artery remains a viable option, up to 30% of patients may not have suitable veins/arteries available due to disease or previous use. As such, synthetic cardiovascular grafts have been developed, with grafts made of materials such as expanded polytetrafluoroethylene (ePTFE) and polyethylene terephthalate (trade marked as Dacron™) emerging over the years. However, the graft technology available today has not fully met clinical needs and there is still room for improvement regarding the low patency of grafts due to non-compliance and the inclination towards thrombogenesis and intimal hyperplasia (IH) [3,4]. This is especially seen in small diameter coronary bypass grafts as Dacron™ is usually used in large diameter grafts and both Dacron™ and ePTFE exhibit low patency for small diameter grafts [5]. When the surface of the implanted graft comes into contact with the surrounding bio-environment such as proteins in the extracellular matrix (ECM), blood cells and endothelial cells, cascades that lead to thrombosis will be triggered. In addition, intimal hyperplasia, which is due to hyperplasia of tissues surrounding the implant will also occur, which will lead to outcomes such as the narrowing of the blood vessel lumen. As such, there is thriving research surrounding the construction of implants that can prevent such processes [3].

**Figure 1:**
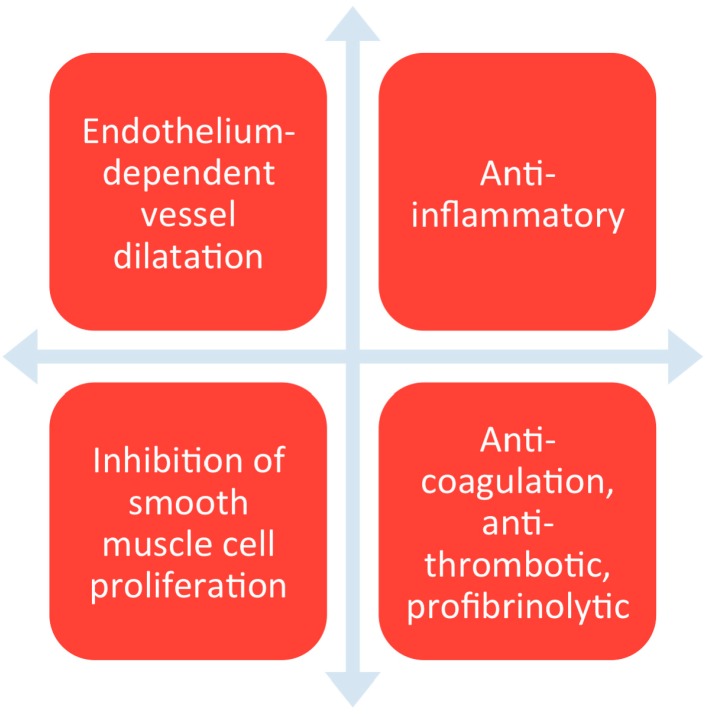
Shows the different functions of endothelium.

If we look at the natural lining of blood vessels, the endothelium prevents thrombosis and IH through various mechanisms [6]. These include the action of molecules like thrombomodulin, tissue factor pathway inhibitor, heparan sulfate proteoglycan, and through the release of prostacyclin and nitric oxide (NO). The endothelium also plays a strong role in controlling diameter of blood vessels via NO-induced pathways. The endothelium also regulates inflammation and thrombosis, both factors leading to IH [7]. As such, due to the remarkable functionality of the endothelium (Figure 1), research in recent years has been directed at two approaches: mimicking the characteristics of the endothelium on the surface of grafts and inducing the endothelialisation of the graft surface itself.

Research has shown that *in vitro* endothelialisation of grafts can improve their long term patency and prevent thrombogenesis [8,9] and this is improved by applying a biomimetic matrix of adhesive cells and growth factors that result in better EC seeding results, with the ECs adopting their normal phenotype [10]. However, *in vitro* endothelialisation of grafts involve multiple specialized procedures (obtaining cells, implanting cells *etc.*) and the cell cultures require a substantial incubation period so it can be seen as cost ineffective, inconvenient and limited to well-equipped facilities.

Therefore, there has recently been an increased interest in nanofabricated cardiac grafts enhanced with biomaterials that can promote *in situ* endothelialisation without IH and thrombosis occurring during endothelium formation. This review aims to look at developments over the recent years to promote the *in situ* endothelialisation of cardiovascular grafts and subsequently improve their patency.

## 2. Materials for Graft Construction

Materials used for cardiovascular graft construction can vary. Non-biodegradable, synthetic materials include ePTFE, Dacron™ and polyurethane. Both ePTFE and Dacron™ have largely similar rates of patency [11]. Polyurethane on the other hand is a copolymer consisting of a hard domain, a chain extender and a soft domain. Common medical-grade polyurethanes have soft domains based on polyester, polyether or polycarbonate. For instance, a poly(carbonate-urea)urethane vascular graft has been developed which showed compliance similar to human arteries [12].

Biodegradable scaffolds are another option being explored by researchers. Examples of biodegradable polymers incorporated into graft design include polyglycolic acid (PGA), polyhydroxyalkanoates, or polycaprolactone (PCL). Biodegradable polymer constructs allow for the spatial and temporal release of substances to enhance endothelialisation, but drawbacks with regards to their long-term patency still exist.

In addition, research has also been done into the manipulation of native ECM proteins such as collagen and fibrin into graft scaffolds. At the same time, decellularized allogeneic or xenogenic grafts have been used as scaffolds. A review in 2010 by Ravi and Chaikof describes various biomaterials used for vascular tissue engineering, along with some of the pros and cons that come with using certain materials [13]. Many approaches that show promise combine biological components to increase haemocompatibility and synthetic components to improve stability, incorporating the best of both worlds.

## 3. Potential Recruits for *in Situ* Endothelialisation

ECs and endothelial progenitor cells (EPCs) all have the potential to be recruited onto an implanted graft for endothelialisation. Besides seeding the graft with ECs, ECs can also proliferate from the site of anastomosis of the graft and the neighbouring vessel wall as well as grow into the graft surface through pores together with capillaries and as such neighbouring ECs play an important role in regenerating vascular structures [14,15]. That being said, ECs usually only grow 1–2 cm into the graft from the site of anastomosis and hence cannot fully endothelialise the graft [4].

EPCs can be found in circulation and can be captured, adhere, proliferate and differentiate into ECs at graft surfaces. They respond to factors such as haemodynamics and chemical stimuli at target sites, which is important for the healing and recellularization of implants [16]. The cell surface markers for human EPCs are reported to include CD34^+^, Tie-2^+^, CD31^+^, UEA-1^+^, ac-LDL^+^, CD45^+^, vascular endothelial growth factor receptor-2 (VEGFR-2), and von Willebrand factor (vWF); these markers contribute to cell adhesion, vascular permeability and other reactions during neovascularisation [17]. Recent work has suggested that putative EPCs consist of different subpopulations and types (early and late outgrowth) and endothelial colony forming cells (ECFC) are the only circulating cells that have the properties of the originally defined EPCs [18]. Late outgrowth cells are the ones that are proposed to end up as vascular structures eventually. One model proposed for vascular healing is that pro-angiogenic haematopoietic cells (PACs), which are not actual endothelial progenitors, act as paracrine activators which recruit and cooperate with ECFC for vascular repair and regeneration [18,19]. These PACs exhibit the markers such as CD34^+^, CD133^+^, CD45^+^, CD31^+^, CD14^−^, and CD235a^−^ [20] some of which are similar to that exhibited by ECFCs, and while they form a synergistic relationship with ECFCs to promote vascularisation, they do not actually become vascular structures and their fate is not specified to endothelium [21]. While there is no definitive conclusion as to the exact mechanism of how EPCs and ECs contribute to re-endothelialisation, studies show that incorporation of EPCs (without a definitive classification currently available, we shall refer to the subpopulations as generic early and late EPCs) still has a substantial potential to improve vascular function, and hence recruitment of EPCs is crucial for long-term graft patency [22]. The design of cardiovascular grafts must take into account methods to home in and mobilize EPCs, and induce their adhesion and differentiation into ECs.

## 4. Endothelial Progenitor Cells: Mobilization

EPC levels in circulation are considered relatively low, especially in the patients who need bypass grafts and tend to be of an advanced age [23]. As such, to improve endothelialisation of implanted grafts, certain strategies have to be implemented to increase the number of circulating EPCs [24] and for EPC homing to targeted sites.

Several factors, namely vascular endothelial growth factor (VEGF) [25], stromal cell-derived factor-1 (SDF-1) [26], granulocyte-colony stimulating factor (G-CSF) [27], HMG-CoA reductase inhibitors (statins) [28], peroxisome proliferator activated receptor-gamma (PPAR-γ) agonists [29], erythropoietin (EPO) [30], nerve growth factor (NGF) [31], brain-derived neurotrophic factor (BDNF) [32], angiotensin II antagonists (e.g., Irbesartan) [33] and certain CXCR4 antagonists [34] have been identified to increase the mobilization of EPCs. Table 1 shows a summary of recent research on utilization of some of these factors for graft endothelialisation purposes. These factors can be incorporated into the grafts using biomaterials for controlled release or administered via exogenous treatments. It seems reasonable to suggest that incorporating the factors into grafts will allow for a more targeted and sustained effect compared to exogenous treatments like oral administration. An interesting development in recent years is the potential of BDNF to increase graft patency [32]. During neovascularization, early EPCs are mobilized to the targeted site of vascular formation and exhibit paracrine functions, secreting cytokines such as VEGF, interleukin-8, fibroblast growth factors *etc.* These cytokines enhance homing of EPCs and promote angiogenesis. Late EPCs on the other hand, continue to proliferate and differentiate into endothelial cells, forming new vascular structures [35]. BDNF has been found to enhance the ability of early EPCs to secrete VEGF and hence enhance their paracrine function. Early EPCs are also able to form more single clone colonies. Late EPCs are induced by BDNF to proliferate and migrate, as well as differentiate into ECs. In murine models, BDNF-modified tissue-engineered blood vessels (TEBVs) show enhanced endothelialisation and less IH, therefore improving the patency of the TEBV [32].

However, not all research findings show optimistic results for factors like VEGF and SDF-1. For instance, there has been evidence to show that VEGF, while being known as an enhancing factor for endothelialisation, can actually contribute to IH [36]. As for SDF-1, SDF-1 has SMPC recruitment properties (see Table 1), prompting suggestions that it is effective for vascular regeneration due to its ability to recruit EPC as well as SMPC. However, research has also shown that SDF-1 is also associated with IH [37]. Thus it seems reasonable to suggest that more research is required to analyse the interactions of the factors discussed above with other components of the bioenvironment and their pharmacokinetics to allow for controlled utilization in tissue engineering.

**Table 1:** Shows recent research on several examples of factors contributing to EPC mobilization. Key: EPO, erythropoietin; EPC, endothelial progenitor cell; SDF-1, stromal cell-derived factor-1; SMPC, smooth muscle progenitor cell; G-CSF, granulocyte-colony stimulating factor; NGF, nerve growth factor; TEBV, tissue-engineered blood vessel; BDNF, brain-derived neurotrophic factor; VEGF, vascular endothelial growth factor; PLLA, poly(l-lactide); PCL, poly(ε-caprolactone); PPAR-γ, peroxisome proliferator activated receptor-gamma.

Factor	Application	Model	Outcome/Effects
EPO [38]	Myocardial infarction was induced in wild-type mice and EPCs with or without EPO were introduced into myocardium around the infarct.	Murine	Enhanced transplanted EPC survival and improved EPC mobilization.
SDF-1 [39]	SDF-1α was fixed onto heparin, which was conjugated onto microfibrous vascular grafts.	Murine	Increased recruitment of EPCs. Also recruited SMPCs.
G-CSF [40]	Heparin-immobilized, decellularized grafts were implanted and subcutaneous injections introduced to subjects.	Murine	EPCs increased and endothelialisation enhanced. Significantly smaller hyperplastic neointima area.
HMG-CoA reductase inhibitors (e.g., Atorvastatin) [41]	Subjects orally administered atorvastatin.	Murine	Circulating EPCs increased, angiogenesis induced and functional recovery improved.
Angiotensin II antagonists [33]	Subjects treated with irbesartan.	Hypertensive-hypercholestrolaemic hamster	EPC mobilization increased.
NGF [42]	Human mononuclear cells isolated and cultured with NGF. CD133^+^ progenitor cells were incubated with NGF and injected into mice with carotid artery injury. NGF treated TEBV implanted into injured mice.	*In vitro* & murine	*In vitro*, human EPCs form more colonies, are stimulated to differentiate into ECs and show improved migration. *In vivo*, mice EPC show improved mobilization and homing and TEBV endothelialisation enhanced.
BDNF [32]	*In vitro*, BDNF introduced to early and late outgrowth EPCs. BDNF-modified TEBV introduced to murine model.	*In vitro* & murine	*In vitro*, BDNF shows ability to enhance single clone formation and paracrine functions of EPCs. BDNF also helps late EPCs proliferate, migrate and differentiate. *In vivo*, TEBV shows greater endothelialisation than control.
VEGF	Covalent immobilization of VEGF onto surfaces of PLLA and PCL.	*In vitro*	Functionalization process created. VEGF known to increase number of EPCs [25].
PPAR-γ agonist [43]	Endothelial progenitor cells from rat bone marrow were cultured with pioglitazone, a PPAR-γ agonist.	*In vitro*	Apoptosis of EPCs reduced.

## 5. EPC and EC Adhesion and Proliferation

### 5.1. Biofunctionalization of Graft Surfaces to Enhance EPC and EC Adhesion and Proliferation

*In situ* endothelialisation of implanted grafts requires ECs and EPCs to adhere and proliferate on the graft surface. To aid in these processes, several factors have to be taken into consideration. As mentioned earlier, there are several surface markers that are characteristic of EPCs. These markers can be utilized to develop strategies for recruiting the EPCs to the graft surface. Besides that, the surrounding bioenvironment of the cells have to be taken into consideration as well. *In vivo* cells are in contact with the ECM, which consists of an extensive network of proteins, glycoproteins and proteoglycans. Besides providing structural support, the ECM is also important in providing the suitable signals and environment for the adhesion and migration of EPCs via integrins, endothelial cell differentiation [44], and also the modulation of growth factors and cytokines to provide a suitable environment for endothelialisation over longer periods [45].

In this section we will look at strategies used to target the cell surface markers and mimic the ECM to enhance endothelialisation, by supporting EC adhesion, proliferation and differentiation while inhibiting SMC thrombogenesis.

#### 5.1.1. Techniques to Biofunctionalize Graft Surfaces

##### Surface Adsorption of Molecules

Biofunctional molecules can be added onto a surface by simple adsorption and immersion. Fibronectin and stem cell homing factor SDF-1α were adsorbed onto knitted polyester pre-coated with collagen (commercially available Gelsoft™ and Polymaille^®^ C) in a study by Visscher *et al.* [46] The results showed improved EC coverage and adherence, as well as reduced intimal hyperplasia and thrombotic material aggregation [46]. The disadvantage of this method is that superficial adsorption is not able to control the orientation of the ligands, and may not bind them as efficiently onto surfaces, as Visscher *et al.* [46] also discovered that there may be loss of coating due to high flow rates.

##### Chemical Immobilisation

**Figure 2:**
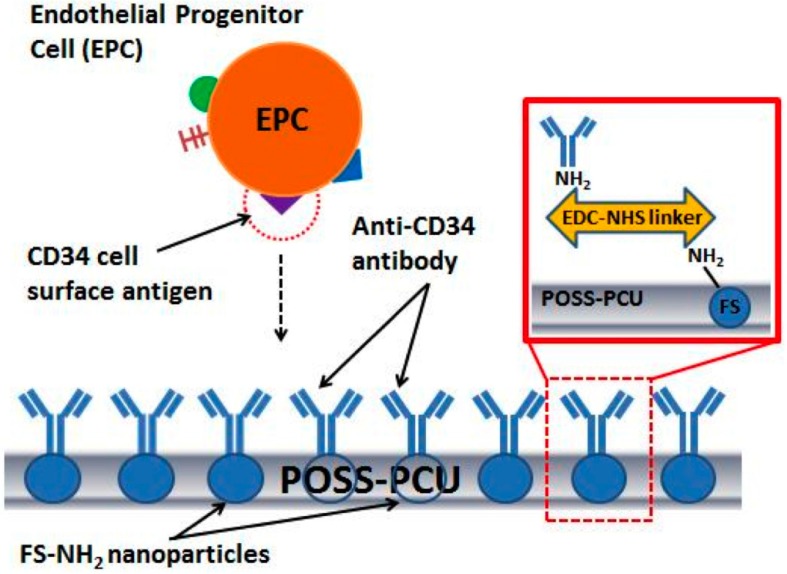
Shows an example of how anti-cd34 antibodies can be immobilized on a polyhedral oligomeric silsesquioxane poly(carbonate-urea) urethane (POSS-PCU) surface. Anti-CD34 antibodies were conjugated with amine-functionalized fumed silica onto POSS-PCU using an *N*-ethyl-*N*'-(3-(dimethylamino)propyl)carbodiimide-*N*-hydroxysuccinimide (EDC-NHS) linker. Reproduced form [50] with permission from Tan *et al.*, copyright 2013.

Covalent linkage allows for control of the distribution and orientation of bioactive molecules on surfaces. It is effective in increasing EC retention, as shown by a group’s enhancement of a poly(carbonate-urea) urethane graft with arginylglycylaspartic acid (RGD peptide) and heparin [47]. Human fibronectin and anti-CD34 conjugated onto the surface of poly-1,8-octanediol-cocitric acid (POC) and poly-l-lactic acid (PLLA) significantly improved EPC adhesion and proliferation in an *in vitro* study [48]. Tan *et al.* [49] conducted a study to provide proof that the concept of immobilizing anti-CD34 antibodies onto polyhedral oligomeric silsequioxane poly(carbonate-urea) urethane (POSS-PCU) to be used as a stent coating can be applied in future cardiovascular implants to enhance endothelialisation. In another study, the same group conjugated anti-CD34 with amine-functionalised fumed silica onto POSS-PCU, which significantly improved *in vitro* haemocompatibility, with decreased platelet adhesion and activation [50] (Figure 2). However, both studies involving POSS-PCU and anti-CD34 have been conducted *in vitro*. Another study further showed that the covalent binding of Laminin type-1 onto porous expanded polytetrafluoroethylene (ePTFE) grafts accelerated neovascularisation and endothelialisation in rats [51]. The actual method of covalent conjugation differs according to the polymer and ligands involved; Bang’s lab published a protocol [52] on various methods to perform covalent coupling with different methods needed for different biomolecules, which some researchers have used as a guide [53]. It is important to find the most appropriate method, as the method of immobilisation has notable effect on the effectiveness of the biomolecule [54].

#### 5.1.2. Targets that Can Be Utilized to Biofunctionalize Graft Surfaces

##### Immobilization of Antibodies onto Graft Surfaces

Methods have been developed to immobilize antibodies that target certain EPC and EC cell markers onto graft surfaces to capture EPCs and ECs from the circulation. For EPCs, various surface markers have been identified that can potentially be used as a target for antibody-immobilized grafts: CD133, kinase insert domain receptor (KDR) and CD34 [55]. As for ECs, markers such as CD31 and KDR have been identified as targets [56,57].

While evidence has shown that anti-CD31 antibodies have the potential to be used to target CD31^+^ ECs for endothelialisation, CD31 is also expressed on lymphocytes, platelets, granulocytes and monocytes [58]. This may lead to unwanted inflammation and immune reactions *in vivo*.

In the past, there were theories that did not consider CD133 as a potential target surface marker for EPCs because of the fact that CD133 is an early haematopoietic stem cell marker and is lost when the stem cells differentiate into more EC-like cells [59]. Hence, CD34 and kinase insert domain receptor (KDR) were thought to be better prospective targets [55]. However, recent evidence shows that CD133 can be more specific than CD34 as a marker for endothelial progenitor cells, and as such, positive results have been shown in animal models when using CD133 as a target for EPC recruitment [60].

CD34 has been used as a target for EPC capture for many studies involving endothelialisation of cardiovascular stents with promising results [50,61,62]. Polymer-free sirolimus-eluting stents coated with anti-CD34 antibodies have been introduced into porcine models, with results showing enhanced endothelialisation and good anti-restenosis potential [62]. Besides that, another group immobilized anti-CD34 antibodies onto POSS-PCU polymer to coat cardiovascular stents (Figure 2) and found that although many of the CD34^+^ cells recruited were not EPCs, the antibody coat increased the haemocompability of POSS-PCU [50]. However, when anti-CD34 antibodies were utilized in synthetic grafts, although endothelialisation increased, IH also increased after a certain period of time [63]. This may be because only a small percentage of circulating CD34^+^ cells are actually EPCs [64], thus non-specific recruitment of CD34^+^ cells may lead to the recruitment of unwanted cells. These cells can also differentiate into cell-types like cardiomyocytes and vascular smooth muscle cells (SMCs) [65]. Thus it seems reasonable to suggest that this may lead to IH as CD34^+^ cells that still have the potential to differentiate into SMCs may be recruited. An alternative is to use a combination of anti-CD34 antibodies and other factors, such as hyaluronan-chitosan, which has shown good recellularization results when applied in stents [66]. Vascular endothelial-cadherin is another example of a potential surface molecule which can reduce the IH problem in CD34^+^ based implants [67]. In both examples, IH has been shown to be less prominent and this could potentially be an improvement instead of using CD34 alone.

KDR, which is also known as VEGF receptor-2 (VEGFR-2) or Flk-1 can be found on both EPCs and ECs. In the study mentioned above (see Table 2), the group managed to improve the human umbilical vein endothelial cell (HUVEC) capture efficiency by changing the orientation of the antibody. This suggests that passive adsorption of antibody may lead to a lower availability of antigen-specific binding sites and specific orientation of the antibodies can lead to better EPC and EC recruitment. While this study has been done using VEGFR-2 as targets, other studies may lend strength to the idea that this concept can be applied to other targets as well. For instance one study showed that the protection of antigen-binding site of antibodies lead to more active sites available [68].

**Table 2:** Recent examples of studies on utilization of antibodies to target cell surface molecules. Key: ePTFE, expanded polyfluorotetraethylene; EPC, endothelial progenitor cell; EC, endothelial cell; KDR, kinase insert domain receptor; VEGFR-2, vascular endothelial growth factor receptor-2; PCL, poly(ε-caprolactone); HUVEC, human umbilical vein endothelial cell.

Target	Application	Model	Outcome
CD133^+^ EPCs [60]	An ePTFE graft with an anti-CD133 antibody multilayer functionalized by heparin/collagen was developed. After being tested for surface modification stability, blood compatibility, haemolysis rate, cellular proliferation and adhesion, *in vivo* testing was carried out as a carotid artery transplant in a porcine model.	Porcine	Endothelialisation onset and rate improved.
CD34^+^ EPCs [63]	ePTFE grafts coated with anti-CD34 antibodies were implanted in 11 pigs between the carotid artery and internal jugular vein.	Porcine	Endothelialisation rate in 72 h increased but IH increased 4 weeks later.
KDR^+^ EPCs and Ecs [57]	Coating of glass coverslips with monoclonal mouse anti-human KDR IgG1 and then incubated with recombinant human KDR/Fc chimera before flow study. Orientation of antibody altered using adsorbed protein G.	*In vitro*	VEGFR-2^+^ HUVECs successfully captured from flow onto solid surface at sub-arterial shear rate. However, when orientation of antibody was altered, 2.5-fold greater capture efficiency observed.

Future possibilities to improve recruitment of EPCs and ECs with antibodies should be focused on using antibodies for different targets together or using antibodies with other biomolecules for greater effectiveness.

##### Immobilization of Proteins and Peptide Sequences onto Graft Surfaces

Over the years, the bio-mimicry of the ECM has inspired the use of ECM protein derived peptide sequences to improve EPC and EC adhesion and proliferation on implant surfaces. Functional domains from ECM proteins such as fibronectin, laminin and collagen type-1 are inspirations for the derivation of such peptides.

The RGD (Arginine-Glycine-Aspartic acid) amino acid sequence can be found on ECM proteins such as fibronectin and laminin. The RGD sequence binds to integrin receptors on cell surfaces (can be found on ECs), which are a family of transmembrane, heterodimeric proteins governing interactions between cells and the ECM proteins and as such can serve as a way to capture integrin-expressing cells [69]. Research has shown that cyclic RGD (cRGD) peptides bind with a greater affinity to ECs as compared to linear RGD peptides since cRGD has a structure more similar to the native ligand of integrins [70]. There is also evidence to show that EPCs express integrins with cRGD binding motifs [71]. A study where RGD tripeptides were incorporated into molecules containing hydrophobic naphthalene groups, Nap-FFGRGD shows greater endothelialisation of the coated grafts [72]. However, it should be noted that RGD binding motifs are not only present on EPCs [73]. Platelets express glycoprotein GPIIb-IIIa on their surface and it recognizes RGD sequences as well [74]. As such, this may lead to unwanted platelet accumulation, causing thrombogenesis and also decrease the ability of the RGD motifs to capture EPCs. Since RGD has relatively low selectivity for EPCs and ECs, it seems reasonable to suggest that by itself, it is insufficient to provide the optimal support for EC and EPC adhesion and it should be modified or used in conjunction with other biofunctional molecules. For instance, in one study, a recombinant fusion protein containing the cellulose-binding domain (CBD)-RGD shows platelet inhibiting ability [75]. Other examples of peptide ligands such as REDV that can be utilized to capture EPCs and ECs are included in Table 3.

**Table 3:** Recent examples of studies on proteins and peptides utilized to improve adhesion and proliferation of ECs and EPCs. Key: EC, endothelial cell; PCL, poly(ε-caprolactone); NO, nitric oxide; TPS, phage display-selected-EPC-specific peptide TPSLEQRTVYAK; EPC, endothelial progenitor cell; PDAM, polydopamine; HUVEC, human umbilical vein endothelial cell; IH, intimal hyperplasia; ELP4, elastin-like polypeptide 4 macromolecules; MAP-RGD, mussel adhesive protein fused with RGD.

Type of Ligand	Protein/Peptide	Method	Model	Outcome/Results
Peptides	cRGD [76]	Transplantation of aortic cRGD-coated self-expanding nitinol stent into rabbit model.	Rabbit	The cRGD peptide was shown to have improved EC adhesion and proliferation.
	Nap-FFGRGD [72]	RGD containing molecule coated onto electrospun biodegradable PCL grafts and the grafts implanted into rabbit carotid arteries.	Rabbit	Increased endothelial coverage, decreased platelet accumulation, and increased smooth muscle remodelling.
	CAG [77]	Electrospun vascular graft constructed containing PCL and CAG and implanted into Sprague-Dawley rats.	Murine	Endothelialisation improved, increased expression of endothelial nitric oxide synthase, lower α-smooth muscle actin.
	REDV [78]	Zwitterionic carboxybetaine methacrylate and butyl methacrylate were copolymerized as coating materials, spin-coated onto substrates, and immobilized with REDV.	*In vitro*	Increased growth of ECs. Decreased accumulation of platelets, limited smooth muscle growth.
	YIGSR [79]	Poly(ethylene glycol) and a diazeniumdiolate NO donor incorporated into polyurethane together with YIGSR peptide sequence.	*In vitro*	Increased EC growth, decreased platelet adhesion.
	TPS [80]	Zwitterionic carboxybetaine methacrylate and TPS incorporated onto electrospun PCL mats.	*In vitro*	Improved hydrophilicity, specifically captures EPCs, decreased platelet adhesion and increased growth of vascular cells.
	PDAM [81]	PDAM coated on 316L stainless steel stents and tested *in vitro*.	*In vitro*	Increased HUVEC adhesion, proliferation, and migration, release of NO, and secretion of prostaglandin I(2). PDAM-modified surface shows ability to decrease the adhesion and proliferation of human umbilical artery smooth muscle cells.
	Collagen and MAP-RGD [82]	PCL scaffolds were first coated in collagen. The collagen-coated scaffolds were then immersed in MAP-RGD solution to immobilize the MAP-RGD. DNA quantification was used to evaluate EC proliferation.	*In vitro*	Highest expression level shown in the PCL/collagen/MAP-RGD group, indicative of improved endothelium sheet formation.
Proteins	Fibronectin [83]	Decellularized rat aortic conduits coated with Alexa488-labelled fibronectin and implanted into Wistar rats for 8 weeks.	Murine	Accelerated endothelialisation but IH occurs after 8 weeks.
	Laminin type-1 [51]	Laminin type-1 is covalently bound to ePTFE grafts and implanted into rats.	Murine	Increased endothelialisation and neovascularization.
	Collagen type-1 with fibronectin [84]	Polystyrene surfaces coated with single and double layers of collagen, fibronectin and collagen + fibronectin.	*In vitro*	Double coating of collagen + fibronectin shows better EC growth.
	ELP4 [85]	ELP4 cross-linked onto polyurethane surface, subjected to reconstituted human blood.	*In vitro*	Enhanced EC adhesion, EC showed organized actin cytoskeleton and enhanced endothelial nitric oxide synthase expression. Decreased platelet adhesion and activation.

Another interesting development is the use of phage display selected peptides. A group managed to select peptide ligands that bind with high specificity and affinity to human blood outgrowth endothelial cells using a combinatorial peptide library and the phage display technique developed in 1985 by Smith [86]. The phage display technique involves the cloning of random-sequenced oligonucleotides in bacteriophage genes to build phage libraries. One such high-affinity, high-specificity peptide ligand is TPSLEQRTVYAK (TPS), which has been used in studies [80]. However, the specific epitope targeted by TPS is still unclear and identification of this epitope can lead to greater success of capture in the future.

One other new technique to utilize adhesion peptides is to incorporate the peptides into the material used to construct the scaffold. In one study, the CAG peptide was used with PCL to create electrospun grafts that were implanted into rats [77]. The results show increased endothelialisation and the potential to inhibit IH.

Regarding ECM proteins, fibronectin, collagen and laminin have been used all along in studies to improve EPC and EC adhesion [44,51,83,84,87]. One other recent development in this area is the use of elastin-like polypeptide 4 macromolecules (ELP4) [85]. ELP4 cross-linked onto polyurethane surfaces showed increased endothelialisation and decreased platelet accumulation.

The use of peptides over large proteins can be advantageous, because there can be other unwanted reactions occurring with the use of large proteins due to the more complex structure compared to peptides. For instance, fibronectin has been shown to induce IH [83]. It has also been shown that natural proteins show less EC adhesion when compared to synthetic RGD and heparin bonded polymers [47]. However, it is also known that some peptides have a lower activity compared to the native protein due to absence of sites that complement or modulate interactions [88]. Hence, research is being done to creating recombinant proteins to solve such issues including incorporating important protein domains into the oligopeptide sequence along with the main binding site [88].

**Figure 3:**
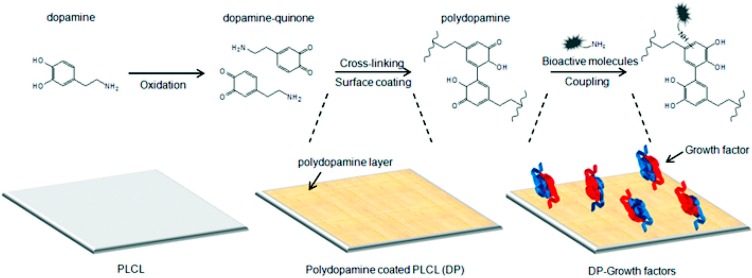
Illustration of method developed by Shin *et al.* [90] to coat PLCL film with PDAM, and mechanism of PDAM capture of VEGF onto the surface. Reproduced from [90] with permission from American Chemical Society, copyright 2012.

In addition, certain proteins and peptides have the ability to capture other biofunctional molecules onto graft surfaces. For instance, mussels have the ability to attach to different surfaces because of dopamine in the adhesive secretions of the animal. Many groups have been inspired by this and have attempted to mimic its properties for example by synthesizing co-polypeptides containing 3,4-dihydroxyphenylalanine (DOPA) and l-lysin [89]. Polydopamine (PDAM) has been used in studies and results show potential to accelerate endothelialisation and inhibit IH [81]. Another study shows that it has the ability to immobilize VEGF (which has pro-endothelialisation properties) when coated onto poly(l-lactide-*co*-ε-caprolactone) (PLCL) film [90] (Figure 3).

In a study by Kang *et al.* [82] collagen and mussel adhesive protein (MAP) fused with RGD were immobilized on PCL scaffolds to investigate its endothelialisation-promoting properties. The scaffolds were loaded into custom-designed perfusion bioreactors. This was done concurrently with investigations into the effect of shear stress preconditioning on endothelialisation enhancement (which will be discussed later). The results show that there was improved endothelium sheet formation (measured via DNA quantification). In addition, the collagen and recombinant MAP-RGD seemed to play a role in reducing thrombogenesis thrombogenesis, as the rounded morphology of platelets seen in the PCL/collagen/MAP-RGD group suggested that they were not activated. However, the authors stated that it was not guaranteed that the MAP-RGD can prevent thrombus formation *in vivo* [82].

##### Immobilization of Nucleic Acid Sequences onto Graft Surfaces

The use of nucleic acid sequences in the biofunctionalization of graft surfaces has garnered increasing interest in recent years. The nucleic acid sequences can be potentially used to improve capture of EPCs and improve endothelialisation.

The functionalization of DNA with certain groups via chemical coupling reactions and the ability to synthesize specially designed DNA strands have resulted in the possibility of incorporating DNA sequences for various uses like drug delivery and more importantly in context of this review, tissue engineering [91]. In fact, a DNA-chitosan complex has been found to be a suitable scaffold material for tissue engineering due to certain characteristics like porosity (which will be discussed later) [92]. In one study, DNA has been used as a biofunctionalized coating on grafts to induce capture of ECs and EPCs with auspicious results [93]. Oligonucleotides were synthesized and labelled with 6-carboxyfluorescein (6FAM) for detection. A 5'-C12-NH_2_ modification was utilized for immobilization. The oligonucleotides were coated on standard ePTFE grafts using a chemical vapour deposition method and were subjected to high shear stress conditions and incubated with human serum, adhesive cells or human blood. The *in vitro* tests results exhibit the ability of the coating to resist high shear stress, improve adhesion of murine EPCs, HUVECs and human ECs, decrease thrombogenicity and increase haemocompatibility [93].

Aptamers are binding molecules comprising nucleic acid sequences with high binding affinities and specificities to certain molecular targets. They are single-stranded DNA or RNA, generated through a process known as Systematic Evolution of Ligands by Exponential Enrichment (SELEX) [94]. They fold into suitable 3-dimensional (3D) structure to bind with the target molecule. In one study, they have been shown to have good potential to be utilized as capture molecules to improve cell recruitment, although in this study osteoblasts were the target capture molecule [95]. In another study, aptamers were immobilized on polyethylene glycol (PEG) hydrogels and tested for cell adhesive properties with good results, suggesting their potential for use in tissue engineering [96].

One group identified, isolated and cultured aptamers with a high affinity for EPCs on polymeric discs with a star-PEG coating. After 10 days, the ability of the porcine EPCs to adhere, proliferate and differentiate was observed *in vitro* and the results of the experiment suggest the use of aptamers to promote endothelialisation and inhibit IH [97]. However, there has also been some evidence showing that aptamers may not be effective. In another study, aptamers targeting CD31^+^ ECs were coated onto aminoparylene-coated stents and compared with non-aptamer-coated stents. The results show that restenosis rates and IH potential were more or less the same for all groups [98].

One of the proposed disadvantages of using oligonucleotides or aptamers is that they can undergo nucleolytic degradation. There are also issues regarding their solubility in aqueous environments [99]. However, studies have shown that they can be chemically modified to be resistant against nuclease degradation [100]. Hence, there is still great potential for their utilization to improve endothelialisation of grafts due to their highly selective properties.

##### Oligosaccharides and Phospholipids

Other groups of biomolecules with the potential to be used to enhance endothelialisation are oligosaccharides and phospholipids. l-selectin, an adhesion receptor found on leukocytes can also be found on EPCs [101]. In one study, the oligosaccharide sialyl Lexis^x^, which has a high affinity for l-selectin, was immobilized onto a collagen matrix and tested *in vitro* and in a murine model [102]. Enhanced endothelialisation properties were observed. It should be noted however that l-selectin is also expressed by leukocytes and as such there may be competitive binding of leukocytes to the oligosaccharide. Besides that, another oligosaccharide worth considering is hyaluronic acid (HA). HA has been utilized in the scaffold material to promote endothelialisation in some studies [103,104], whether being incorporated into the backbone of PU or being used as a surface-modified hydrogel, with promising results. In addition, covalently attached HA onto PU materials in one study has also been shown to support endothelial growth as well as inhibit protein and platelet attachment. However, only when low molecular weight HA was used did the endothelial growth increase [104,105].

A 2-methacryloyloxyethyl phosphorylcholine (MPC) copolymer was immobilized on electrospun poly(ester urethane)urea grafts and tested *in vitro* for platelet adhesion properties. After that, the conduits were implanted into rats. The platelet adhesion was decreased significantly *in vitro* and in the murine models, neointimal tissue formation with both SMC and EC markers was observed [106]. Phosphorylcholine has also been modified with chitosan and used in studies to show adhesion and differentiation of EPCs. Increased EPC adhesion was shown with decreased mesenchymal stem cell adhesion [107].

More research *in vivo* is required to investigate the feasibility of utilizing these relatively uncommon approaches to enhance endothelialisation of cardiovascular grafts.

While most of the studies involving the biofunctionalization of cardiovascular grafts have been done either in the lab or in animal models, one study has shown promising results regarding the effectiveness of biofunctionalization at inducing rapid *in situ* endothelialisation within a simulation of the human cardiovascular system. Biofunctionalized, nanocomposite polymer-based small diameter bypass grafts were connected to a circuit driven by a pump to simulate the pulsatile flow of the cardiovascular system. EPCs were introduced into the circuit and the results showed that after 10 days of incubation, there was a significant increase in the degree of endothelialisation as compared to conventional PTFE grafts used as controls [108]. As such, biofunctionalization shows great potential as a viable method of promoting endothelialisation in cardiovascular grafts.

### 5.2. Magnetic Homing of EPCs and ECs to Site of Endothelialisation

Another potential approach to increase the recruitment of EPCs to graft surfaces is to incorporate magnetic molecules onto target cells and utilize a magnet to attract the targeted cells to the graft surface. There have already been studies done in which target cells coated with magnetic molecules were directed to targeted sites with significant success. In one study, anti-CD34 antibody-coated magnetic nanoparticles were demonstrated to be able to bind to CD34^+^ stem cells *in vivo* while retaining good magnetic properties [109]. In another study, magnetic bionanoparticles acquired from *Magnetospirillum* sp. AMB-1 were used to deliver EPCs to a targeted ischaemic site in a murine model [110]. This shows the potential of the nanoparticles being used to deliver stem cells to implanted synthetic grafts. Besides that, grafts have been designed more recently where iron oxide nanoparticles were used to label EPCs and used to target the labelled cells onto the graft surface, where they attached and proliferated into a continuous endothelium [111]. However, more research needs to be done regarding the consequences of exposure to the magnetic molecules. For instance, a study in 2003 showed that using high concentrations of commercially available magnetic beads, Dynabeads (Life Technologies, Carlsbad, CA, USA), resulted in decreased EC proliferation and metabolism [112].

### 5.3. Micropatterning, Nanopatterning, and the Surface Physical Characteristics of Grafts

The physical and mechanical properties of graft surfaces are also very important in promoting endothelialisation. Stem cells have been shown to respond to topographical cues independent of the surface chemistry [113], and the precise effects of changes in micro- and nano-scale feature geometry (such as gratings, islands and pits) on ECs, SMCs, and fibroblast cells have been described in detail in a recent review by Nazneen *et al.* [114]. As such, surface topography and other physical properties have been taken into account during the development of cardiovascular implants, namely stents [115]. The same concepts can be utilized in the development of graft surfaces and in this section, we will explore some of the characteristics that can be considered in trying to improve the *in situ* endothelialisation properties of cardiovascular grafts.

Regarding manipulating the surface topography, modifications can be done on the micro- or nano-scale. While proponents of nanoscale modulation maintain that it is more relevant due to the similarities in scale to cell receptors and cell interactions with the surroundings [116], proponents of microscale patterning challenge this based on practical reasons [117]. A review by Chong *et al.* [118] summarizes current advances in surface engineering.

Techniques that can modify surface physical characteristics include photolithography, electron beam lithography (EBL), electrospinning (which manipulates nanofibres orientation using an electric field and a rotating drum to collect fibres at different speeds [119]), a form of 3D printing known as microsterolithography (used to modify bone scaffold architecture and internal pore size for enhanced cell proliferation [120]), and ultraviolet (UV) irradiation (which can photofunctionalise surfaces and increase the hydrophilicity of some polymers, such as in the case of the nanocomposite polymer PCU with silsequioxane nano-cages, where adherence and proliferation of HUVECs were improved [121]).

**Figure 4:**
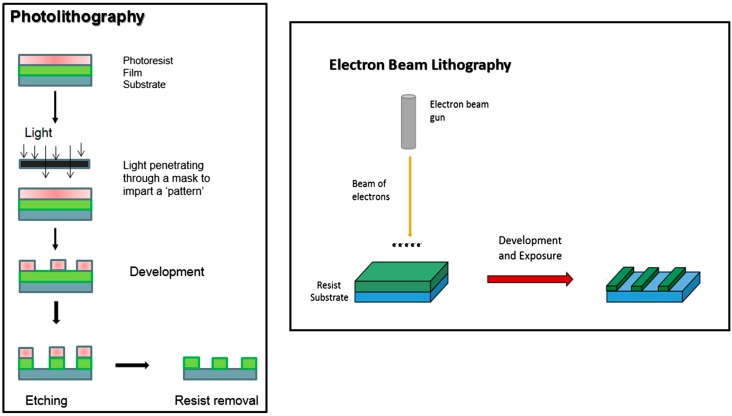
Summarizes the basic principles of photolithography and electron beam lithography [118]. Reproduced form [118] with permission from Elsevier, copyright 2014.

In photolithography, light is used to generate desired patterns on a surface. A light-sensitive polymer (a photoresist) is exposed to UV light through a mask layout. This causes crosslinking, polymerisation or degradation of the photoresist and 2-dimensional (2D) features can be constructed. EBL is an extension of photolithography which uses high-energy electrons instead to create patterns on a nanoscale, unlike photolithography which is used to develop microscale surfaces [122]. Figure 4 below briefly explains the processes of photolithography and EBL.

#### 5.3.1. Electrospun Fibre Diameter and Alignment

Electrospinning is a technique widely used in the production of graft scaffolds [123]. It is therefore important that the structural design of such electrospun grafts be examined regarding their influences on endothelialisation. Many studies have shown that the diameter and the alignment of the fibres used in electrospun grafts have great influence on the growth and metabolism of ECs. In native blood vessels, the medial layer is orientated circumferentially while the intimal layer is oriented longitudinally according to flow [124,125]. As such, efforts have been made to mimic these properties in the design of graft scaffolds. One group created electrospun scaffolds with a composite polymer blend of PCL and collagen type I, varying the degrees of fibre alignment and with a fibre diameter range from 100–1200 nm. After ECs were seeded onto the scaffolds, the results showed that cell alignment increased with greater degree of fibre alignment, and that the cells on aligned scaffolds exhibit robust F-actin bundles running in parallel with the orientated fibres, in addition to high VE-cadherin expression at intercellular junctions. ECs on aligned scaffolds also show less detachment under fluid flow conditions [126]. In another study looking into factors such as fibre diameter, alignment and surface porosity, fibre alignment has also been shown to be able to coax ECs to adopt a more native-like morphology [127]. Besides *in vitro* studies, one group used electrospun poly(l-lactide) (PLLA) scaffolds with controlled fibre alignment and heparin coatings to test cell infiltration in murine models. The results showed that aligned nanofibres can indeed enhance cell infiltration into 3D scaffolds [128]. The different effect fibre diameter (either nanoscale or microscale) has on cell activity may be utilized to create multi-layered scaffolds with better functionality. This has been shown in one study where not only fibre orientation was shown to improve cell alignment, but also that mats with smaller fibre diameters showed less cell infiltration through the surface. As such the group conducting the study has suggested that a bilayer vascular graft surface can be created with the mat consisting of smaller diameter fibres making up the luminal surface and the mat with larger diameter fibres making up the abluminal surface [129].

Thus it can be demonstrated that scaffold fibre diameter and alignment has a significant effect on the activity of ECs. One study even suggested that such features are more important than chemotactic gradients in determining cell motility and hence should be taken into account in graft design [130]. The group used electrospun HA scaffolds under a gradient of VEGF and found out that HUVEC motility was influence mainly by the HA fibre alignment rather than the chemical gradient of VEGF.

#### 5.3.2. Surface Roughness

Numerous studies have shown that surface roughness has an impact on EC adhesion and growth. This aspect has been taken into account for the development of stents and studied. One group compared the *in vitro* endothelialisation of titanium stents with different surface roughness. Flat titanium surfaces, titanium surfaces with depositions of 50 nm and 1 μm thickness were compared. The results showed that the titanium surfaces with submicron roughness exhibited the highest endothelial cell migration, adhesion and integrity along with the highest nitric acid/endothelin-1 ratio and lowest platelet adhesion. Nanometre surface roughness provided better results than flat surfaces in all aspects as well. This shows that nanometre and submicron surface features can potentially be recruited in the design of titanium stents [131]. This characteristic can potentially also be adopted for the development of grafts.

Besides that, Dickenson *et al.* [132] used a SiO_2_ substrate with micropillars and showed that micropillars taller than 3 μm greatly inhibited EC spreading and adhesion. After that, the group also demonstrated that ECFCs and HUVECs preferentially adhered to and showed greater spreading on fibronectin (Fn)-coated micropillar SiO_2_ substrates with micropillar heights of less than 6 μm. The group also showed that the spacing between micropillars and the micropillar diameter have an effect on the extensions expressed by adhered HUVECs, where larger spacing and smaller diameters showed more pronounced adhesive protrusions. In addition, the group also showed that ECFCs only aligned on patterned Fn micropillar arrays with micropillar diameters between 1–2 μm [132]. Hence it seems reasonable to suggest that there is an optimal range regarding topographical feature diameter, spacing and height at which endothelialisation can be optimized.

#### 5.3.3. Porosity of Graft Surfaces

The surface porosity is another important factor to be considered when designing cardiovascular grafts. It has been shown that ECs are unable to bridge pores wider than EC cell diameters and thus ideal pore size should be between 10–45 μm [133]. One study has shown that ePTFE grafts with pore size of 30 μm exhibit the highest rate of EC-like cell growth [134]. In addition, the same study also suggests that the infiltration of perigraft tissue via pores support the growth of the neointima, hence underlying the importance of pores in graft design. In another study, since larger pore sizes are important for cell infiltration but may lead to blood leakage through graft walls, one group has shown that by manipulating pore size in different layers for the luminal and abluminal surface of grafts, tissue regeneration can be optimized with minimal blood leakage [135].

#### 5.3.4. Peptide Patterning

It is important that the physical and chemical modifications of graft surfaces be considered in tandem to improve *in situ* endothelialisation. In one study, it is shown that the spatial patterning of RGD ligands have important effects on whether successful target cell adhesion takes place [136]. Besides that, another study showed the relative importance of topography and RGD ligand density in inducing endothelialisation [137]. The group did this comparison by using either micro-scaled or nano-scaled pyramids with different RGD ligand densities in tests for EC adhesion. The results showed that the size of pyramids are the main factor affecting EC adherence but average RGD ligand density is the predominant factor controlling cell spreading and focal adhesion length. Relatively low RGD densities promote cellular adhesion and spacing while higher densities are important in the formation of focal adhesions and stress fibres in ECs.

### 5.4. Shear Stress Preconditioning

A study conducted by Kang *et al.* [82] which was mentioned earlier, investigated the effects of shear stress preconditioning on endothelial cell proliferation. A perfusion bioreactor was used to simulate physiological shear stress conditions, and the configuration of the perfusion bioreactor system modified to exhibit different flow rates. The test was run for 6.5 days. The conditions were: (A) static; (B) high shear stress (5 mL/min) for 6.5 days; (C) 0.5 mL/min with transient increase to 5 mL/min on day 3 and (D) 0.5 mL/min with transient increase to 5 mL/min on day 6. Under *in vitro* conditions, it was shown that the timing and magnitude of shear stress conditions have an effect on EC proliferation. For conditions where there was a long period of steady laminar flow (B and D), cell proliferation was inhibited. For conditions A and C, there were the highest levels of EC proliferation and adhesion protein expression after 6 days. However, C had higher levels of collagen type IV and fibronectin expression compared to A, which had the lowest out of the four conditions. It can be thus suggested that a transient increase in shear stress at the right time can be utilized in shear stress preconditioning to enhance endothelialisation [82]. The results that laminar shear stress inhibit EC proliferation were also seen in previous studies [138].

## 6. Regulating Differentiation: From Progenitor Cells to Mature Endothelium

EPCs from the circulation need to differentiate into mature ECs on the graft surface before they can form fully functional endothelium. The heterogeneity of EPC subpopulations has shown that some CD34^+^ EPCs can potentially differentiate into SMCs [139]. ECs have different phenotypes, as shown in a study on its *in vitro* growth on bare Ti metal [10] and stainless steel [140] where these materials induced it to become pro-thrombotic. Therefore it is important to correctly control the differentiation of EPCs to healthy ECs. Most research focuses on the capture and adhesion of EPCs, with the assumption that they will automatically differentiate into healthy ECs, or simply note the general effects of improving endothelialisation, without isolating the effects on the different stages (mobilisation, homing, adhesion, proliferation, differentiation). Undifferentiated EPCs have markers including CD34, VEGFR-2, and AC133, and when differentiating to ECs lose CD34 and AC133 while gaining vWf, CD31, VE-cadherin and endothelial nitric oxide synthase (eNOS) [141]. There is a need to further research and understand the cell biology of EPCs and their differentiation pathways. The material and surface topography of the grafts can affect the differentiation, as can soluble signalling biomolecules present in the physiological environment.

### 6.1. Materials and Topography of Grafts

The implant material alone can influence the differentiation of EPCs. A group demonstrated this with titanium surfaces of varying hydrophilicity and roughness, comparing the markers produced (which can indicate degree of differentiation) with controls of bare and fibronectin-coated biocompatible plastic [142]. Another study found that EC differentiation of adipocyte-derived stem cells (ADSC) is enhanced by nanograted surfaces, through the upregulation of the expression of relevant markers [143]. Although ECs would not differentiate from ADSCs *in vivo*, the study nevertheless conveys the significance of surface topography for endothelial cell differentiation.

### 6.2. Utilization of Signalling Biomolecules

Chemokines, cytokines and growth factors all have varying contributions to the process of endothelialisation, not only in promoting EPC differentiation to EC but also the number of circulating EPCs and their proliferation. VEGF [144], G-CSF, endothelial cell growth factor (ECGF) [145], stromal cell derived factor 1 [70], cysteine-rich 61 [146], erythropoietin [30] and interleukin-8 [147], among others, have been shown to have a role in vascular repair and EC differentiation. In the complex physiological environment, there may be interactions between these signalling biomolecules that could result in unintended effects. The broad effects and the natural role of these molecules in inflammation may produce undesired results such as IH, which must be kept in mind when considering the potential of these molecules in regulating EPC differentiation.

### 6.3. Shear Stress Acting on Graft Surfaces

Shear stress is naturally present *in vivo* but has been included here as it has been shown to induce EPC differentiation [148,149], and must be taken into account in graft design and also during *in vitro* testing. Studies have discussed different pathways and mechanisms but the overall effect is consensual. One group explained that shear stress activates mechanosensitive molecules such as integrin β_1_, to cause cytoskeletal rearrangement [150]. Akt (protein kinase B) activation appears to be involved [151], with varying mechanisms. A signal pathway Flk-1–PI3K–Akt–HDAC3–p53–p21 was proposed with the activation of histone deacetylase, with a similar one for the activation of VEGF-R2 in the differentiation of EC from stem cells [152]. Shear stress has also been shown to inhibit EPC differentiation towards SMC, and hence direct it towards development into healthy ECs [153]. In addition, Morgan *et al.* [154] discovered that shear stress has a synergistic relationship with topographical cues in promoting EC adhesion as well, highlighting the importance of considering shear stress and physiological conditions in future studies.

## 7. Preventing Thrombogenesis, IH Formation and Inflammation during Endothelialisation

A great deal of attention has been placed on accelerating the process of endothelialisation, yet there will inevitably be a window period between implantation and the formation of a fully functional endothelium. Within this time it is essential to ensure that the graft is antithrombotic and anti-IH while simultaneously attracting EPCs. Grafts that may be good for adhesion of EPCs might also attract platelet aggregation and stimulate SMC activity. The grafts need to stimulate the required processes while inhibiting the undesirable ones. This can be achieved using methods such as anticoagulants like heparin. Heparin has been shown to improve haemocompatibility while promoting *in vivo* endothelialisation [155]; it has also been shown to improve EPC adhesion, proliferation and differentiation into the healthy EC phenotype while decreasing SMC proliferation in an *in vitro* experiment [156].

One other method of interest is the utilization of NO. One approach is to create surface catalytic sites that can react with endogenous NO precursors such as *S*-nitrosothiols to generate NO. The supply of NO is thus from endogenous sources and potentially unlimited [157]. This is in contrast with NO being stored in a polymer reservoir or immobilized as a precursor or donor, to improve haemocompatibility by its *in situ* release [158,159]. It is more difficult to achieve a sustained long-term release at physiological levels with the latter method [160]. In one study by de Mel *et al.* [161] *S*-nitrosothiols (*S*-Nitroso-*N*-acetylpenicillamine (SNAP) and *S*-Nitrosoglutathione (GSNO)) were incorporated into POSS-PCU coronary artery bypass grafts and subjected to physiological conditions. Optimal NO elution was shown with 2% SNAP-POSS-PCU. The NO released demonstrated anti-thrombogenic properties as well as greater adhesion of EPCs and reduced platelet adhesion [161].

## 8. Conclusions and Future Directions

Of the various methods of endothelialisation, an *in vivo*, pro-healing approach is the most promising. It can potentially address the clinical need for implants that can be used “straight out of the box”, as it uses the human body’s capacity to heal, without requiring any surgical harvesting of patient-specific tissues.

Many different strategies to enhance this *in situ* endothelialisation have been studied, with focus on supporting the mobilisation, homing, adhesion of EPC from the bloodstream to the graft surface, and their proliferation and differentiation into mature ECs, to form a fully functioning haemocompatible endothelium capable of resisting thrombosis and restenosis due to intimal hyperplasia, even in small diameter grafts.

The ideal implant needs to be able to increase the number of EPCs in the blood and aid their homing to the actual graft surface where they are needed. It needs to promote selective adhesion of EPCs and ECs onto its surface while inhibiting thrombogenesis, IH and inflammation with suitable surface nanotopography. This can be controlled with various methods of material fabrication, including modification of the physicochemical surface and also by surface coatings. Once adhered, the implant should direct the proliferation and differentiation of EPCs to suitable healthy phenotypes of EC. Adequate porosity can support angiogenesis and the growth of sub-endothelial tissues.

This is a massive, dynamic and constantly evolving field with lots of promising research. The challenge is to combine various strategies to create the ideal graft to cater to the cardiovascular clinical needs of the modern day, with suitable experiments and clinical trials, and to refine the fabrication technique to enable cost-effective mass production.
